# Irisin Stimulates the Release of CXCL1 From Differentiating Human Subcutaneous and Deep-Neck Derived Adipocytes *via* Upregulation of NFκB Pathway

**DOI:** 10.3389/fcell.2021.737872

**Published:** 2021-10-11

**Authors:** Abhirup Shaw, Beáta B. Tóth, Róbert Király, Rini Arianti, István Csomós, Szilárd Póliska, Attila Vámos, Ilma R. Korponay-Szabó, Zsolt Bacso, Ferenc Győry, László Fésüs, Endre Kristóf

**Affiliations:** ^1^Laboratory of Cell Biochemistry, Department of Biochemistry and Molecular Biology, Faculty of Medicine, University of Debrecen, Debrecen, Hungary; ^2^Doctoral School of Molecular Cell and Immune Biology, University of Debrecen, Debrecen, Hungary; ^3^Department of Biophysics and Cell Biology, Faculty of Medicine, University of Debrecen, Debrecen, Hungary; ^4^Genomic Medicine and Bioinformatics Core Facility, Department of Biochemistry and Molecular Biology, Faculty of Medicine, University of Debrecen, Debrecen, Hungary; ^5^Department of Pediatrics, Faculty of Medicine, University of Debrecen, Debrecen, Hungary; ^6^Faculty of Pharmacy, University of Debrecen, Debrecen, Hungary; ^7^Department of Surgery, Faculty of Medicine, University of Debrecen, Debrecen, Hungary

**Keywords:** obesity, adipose tissue, irisin, cytokines, CXCL1, integrins, NFκB, angiogenesis

## Abstract

Thermogenic brown and beige adipocytes might open up new strategies in combating obesity. Recent studies in rodents and humans have indicated that these adipocytes release cytokines, termed “batokines”. Irisin was discovered as a polypeptide regulator of beige adipocytes released by myocytes, primarily during exercise. We performed global RNA sequencing on adipocytes derived from human subcutaneous and deep-neck precursors, which were differentiated in the presence or absence of irisin. Irisin did not exert an effect on the expression of characteristic thermogenic genes, while upregulated genes belonging to various cytokine signaling pathways. Out of the several upregulated cytokines, *CXCL1*, the highest upregulated, was released throughout the entire differentiation period, and predominantly by differentiated adipocytes. Deep-neck area tissue biopsies also showed a significant release of CXCL1 during 24 h irisin treatment. Gene expression data indicated upregulation of the NFκB pathway upon irisin treatment, which was validated by an increase of p50 and decrease of IκBα protein level, respectively. Continuous blocking of the NFκB pathway, using a cell permeable inhibitor of NFκB nuclear translocation, significantly reduced CXCL1 release. The released CXCL1 exerted a positive effect on the adhesion of endothelial cells. Together, our findings demonstrate that irisin stimulates the release of a novel adipokine, CXCL1, *via* upregulation of NFκB pathway in neck area derived adipocytes, which might play an important role in improving tissue vascularization.

## Introduction

Recent studies indicated the presence of thermogenic adipose tissue, capable of dissipating energy as heat under sub-thermal conditions in healthy human adults (Cypess et al., 2009; Leitner et al., 2017). These are located in cervical, supraclavicular, axillary, mediastinal, paravertebral, and abdominal depots (Saito et al., 2009; van Marken Lichtenbelt et al., 2009; Virtanen et al., 2009); supraclavicular, deep-neck (DN), and paravertebral having the highest amounts. Together these depots account for 5% of basal metabolic rate in adults, highlighting their importance in combating obesity and type 2 diabetes mellitus (van Marken Lichtenbelt and Schrauwen, 2011). In rodents, these thermogenic adipocytes are either classical brown or beige depending on their origin and distribution (Rosen and Spiegelman, 2014; Kajimura et al., 2015). In addition to their role in thermogenesis, these adipocytes also secrete adipokines, termed “batokines”, which have been shown to exert autocrine, paracrine, or endocrine activity (Villarroya et al., 2017). For example, vascular endothelial growth factor A (VEGF-A) secreted by brown adipocytes promotes angiogenesis and vascularization of brown adipose tissue (BAT) (Xue et al., 2009; Sun et al., 2014; Mahdaviani et al., 2016), while Fibroblast growth factor (FGF) 21 enhances the beiging of white adipose tissue (WAT) in animal studies (Cuevas-Ramos et al., 2019) and increases thermogenesis in BAT (Hondares et al., 2011; Wang et al., 2015; Ruan et al., 2018). Understanding the roles of batokines in the human body is an area of active research (Villarroya et al., 2019; Ahmad et al., 2021).

Irisin, a cleaved product of the transmembrane protein Fibronectin Type III domain-containing protein 5 (FNDC5), was discovered as a myokine in mice and was shown to be a browning inducing endocrine hormone (Boström et al., 2012; Zhang et al., 2014), presumably acting *via* integrin receptors (Kim et al., 2018). A recent publication has shown that individuals with obesity exhibited a downregulation of *FNDC5* gene and protein expression in visceral and subcutaneous fat depots (Frühbeck et al., 2020). In mice, irisin secretion was induced by physical exercise and shivering of skeletal myocytes, which induced a beige differentiation program in subcutaneous WAT (Boström et al., 2012). In rats, irisin was also found to be released from cardiomyocytes at much higher amount than skeletal muscles (Aydin et al., 2014). Lower levels of circulating irisin was observed in patients with cardiovascular disease (Polyzos et al., 2018). Irisin has also been shown to improve cardiac function and inhibit pressure overload induced cardiac hypertrophy and fibrosis (Yu et al., 2019). In humans, inconsistent effects were found when adipocytes of different anatomical origins were treated with recombinant irisin (Raschke et al., 2013; Lee et al., 2014; Silva et al., 2014; Kristóf et al., 2015; Klusóczki et al., 2019; Li et al., 2019). How irisin affects the differentiation of the thermogenically prone neck area adipocytes still awaits description. We have previously reported that human DN adipose tissue biopsies released significantly higher amounts of interleukin (IL)-6, IL-8, monocyte chemoattractant protein 1 (MCP1) as compared to subcutaneous ones, which was further enhanced upon irisin treatment (Kristóf et al., 2019).

C-X-C Motif Chemokine Ligand (CXCL) 1, previously known as growth-related oncogene (GRO)-α, is a small peptide belonging to the CXC chemokine family. Newly synthetized CXCL1 by vessel-associated endothelial cells and pericytes facilitates the process of neutrophil diapedesis (Gillitzer and Goebeler, 2001). A recent study showed that the chemokine CXCL14 is secreted by BAT under thermogenic stimulation, which induces browning of WAT by recruitment and activation of M2-macrophages (Cereijo et al., 2018). This study reinforced the fact that chemokines play an important role in thermogenic activation.

In this study, we aimed to get an overview of all the genes in which expression is regulated by irisin. For this, we have performed a global RNA-Sequencing comprising of *ex vivo* differentiated adipocytes of subcutaneous and deep depots of human neck from nine individuals and analyzed the upregulated genes upon irisin treatment. Surprisingly, several genes which encode secreted proteins were upregulated. Out of those, CXCL1 was found to be the highest expressed and a novel adipokine induced in differentiating adipocytes of both origins. The CXCL1 release was stimulated partially *via* the upregulation of nuclear factor-κB (NFκB) pathway. We found that the secreted CXCL1 had an adhesion promoting effect on endothelial cells, supporting that irisin can exert effects not directly linked to heat production.

## Materials and Methods

### Materials

All chemicals were obtained from Sigma Aldrich (Munich, Germany) unless otherwise stated.

### Isolation, Cell Culture, Differentiation, and Treatment of hASCs

Human adipose-derived stromal cells (hASCs) were obtained from stromal-vascular fractions of subcutaneous neck (SC) and DN tissues of volunteers, aged between 35–75 years, undergoing planned surgical treatment. A pair of biopsies from SC and DN areas was obtained from the same donor, to avoid inter-individual variations (Sárvári et al., 2015; Kristóf et al., 2019; Tóth et al., 2020). Patients with known diabetes, body mass index > 30, malignant tumor, infection or with abnormal thyroid hormone levels at the time of surgery were excluded from the study. Written informed consent was obtained from all participants before the surgery. Data of the donors included in RNA-sequencing are listed in Supplementary Table 1.

hASCs were isolated and cultivated as previously described (Sárvári et al., 2015; Kristóf et al., 2019; Tóth et al., 2020). The absence of mycoplasma was confirmed by PCR analysis (PCR Mycoplasma Test Kit I/C, Promocell, Heidelberg, Germany). Cells were differentiated following a previously described white adipogenic differentiation protocol, with or without the addition of human recombinant irisin (Cayman Chemicals, MI, United States) (provided in 50 mM Tris pH 8.0, 150 mM sodium chloride, and 20% glycerol stocks) at 250 ng/mL (20 nM) concentration (the stock was diluted 1:6,500) (Fischer-Posovszky et al., 2008; Raschke et al., 2013; Kristóf et al., 2019). Media were changed every 4 days and cells were collected after 14 days of differentiation. In every repetition, untreated and irisin treated samples were obtained from the same donor. Cells were incubated at 5% CO_2_ and 37°C. Where indicated, cells were treated with RGDS peptide (10 μg/mL, R&D systems, MN, United States) (Kim et al., 2018) or SN50 (50 μg/mL, Med Chem Express, NJ, United States) (Sárvári et al., 2015).

### RNA Isolation, RT-qPCR, and RNA-Sequencing

Cells were collected in Trizol reagent (Thermo Fisher Scientific, MA, United States) and RNA was isolated manually by chloroform extraction and isopropanol precipitation. To obtain global transcriptome data, high throughput mRNA sequencing was performed on Illumina Sequencing platform (Tóth et al., 2020). Total RNA sample quality was checked by Agilent Bioanalyzer using Eukaryotic Total RNA Nano Kit; samples with RNA integrity number >7 were used to prepare the library. Libraries were prepared by NEBNext^®^ Ultra^TM^ II RNA Library Prep for Illumina (New England BioLabs, Ipswich, MA, United States). Sequencing runs were executed on Illumina NextSeq500 using single-end 75 cycles sequencing. The reads were aligned to the GRCh38 reference genome (with EnsEMBL 95 annotation) using STAR aligner (Dobin et al., 2013). To quantify the reads, featureCounts was used (Liao et al., 2014). Gene expression analysis was performed using R. Genes with very low expression and with outlier values were removed from further analysis. To further remove outlier genes, Cook’s distance was calculated and genes with Cook’s distance higher than 1 were filtered out. PCA analysis did not show any batch effect considering sequencing date and the donor origin, sex or tissue origin (data not shown). DESeq2 algorithm was used to detect the differentially expressed genes based on adjusted *p* values < 0.05 and log2 fold change threshold >0.85. Grouping was performed based on Panther Reactome pathways^1^. Heatmap visualization was performed on the Morpheus web tool^2^ using Pearson correlation of rows and complete linkage based on calculated z-score of DESeq normalized data after log_2_ transformation (Tóth et al., 2020). The interaction networks were determined using STRING^3^ and constructed using Gephi 0.9.2^4^. The size of the nodes was determined based on fold change (Tóth et al., 2020).

For RT-PCR, RNA quality was evaluated by spectrophotometry and cDNA was generated by TaqMan reverse transcription reagents kit (Thermo Fisher Scientific) followed by qPCR analysis (Szatmári-Tóth et al., 2020). LightCycler 480 (Roche Diagnostics, IN, United States) was used to determine the normalized gene expression using the probes (Applied Biosystems, MA, United States) which are listed in Supplementary Table 2. Human *GAPDH* was used as an endogenous control. Samples were run in triplicate and gene expression values were calculated by the comparative cycle threshold (Ct) method. ΔCt represents the Ct of target after deducting the *GAPDH*. Normalized gene expression levels were calculated by 2^–ΔCt^.

### Antibodies and Immunoblotting

Samples were collected, separated by SDS-PAGE, and transferred to PVDF Immobilon-P transfer membrane (Merck-Millipore, Darmstadt, Germany) as previously described (Szatmári-Tóth et al., 2020). The following primary antibodies were used overnight in 1% skimmed milk solution: anti-p50 (1:1,000, 13755, Cayman Chemicals), anti- IκBα (1:1,000, 4812, Cell Signaling Technology, MA, United States), and anti-β-actin (1:5,000, A2066, Novus Biologicals, CO, United States). HRP-conjugated goat anti-rabbit (1:10,000, Advansta, CA, United States, R-05072-500) or anti-mouse (1:5,000, Advansta, R-05071-500) IgG were used as secondary antibodies, respectively. Immobilion western chemiluminescence substrate (Merck-Millipore) was used to visualize the immunoreactive proteins. FIJI was used for densitometry.

### Immunostaining Analysis and Image Analysis

hASCs from SC and DN areas were plated and differentiated in eight well Ibidi μ-chambers (Ibidi GmbH, Gräfelfing, Germany). Cells were treated with Brefeldin A (100 ng/mL), an inhibitor of intracellular protein transport, 24 h prior collection to sequester the released CXCL1 (Sárvári et al., 2015; Kristóf et al., 2019). After that, cells were washed with PBS, fixed by 4% paraformaldehyde, permeabilized with 0.1% saponin and blocked by 5% milk as per described protocols (Szatmári-Tóth et al., 2020). The cells were incubated subsequently with anti-CXCL1 primary antibody (1:100, 712317, Thermo Fisher Scientific) and Alexa 488 goat anti-rabbit IgG (1:1,000, A11034, Thermo Fischer Scientific) secondary antibody for 12 and 3 h at room temperature, respectively. Propidium iodide (1.5 μg/mL, 1 h) was used to label the nuclei. A secondary antibody test was also performed where the cells were incubated only with the respective secondary antibodies. Images were acquired with Olympus FluoView 1000 confocal microscope and analyzed by FIJI (Szatmári-Tóth et al., 2020). Boundaries of preadipocytes and differentiated adipocytes were identified manually based on brightfield (BF) images and nuclear staining, followed by quantification of immunostaining intensity. Adipogenic differentiation rate was quantified as described previously (Doan-Xuan et al., 2013; Kristóf et al., 2015).

### Determination of the Released Factors

Supernatants of samples from cell culture experiments were collected at regular replacement of media, on days 4, 12, 18, 21 of differentiation, wherever indicated. For SC and DN, supernatants were collected and stored at −20°C from the differentiated cells of the same donor and considered as one repetition, followed by repetition with subsequent donors. For tissues, 10–20 mg of SC and DN tissue samples from the same donor were floated for 24 h in DMEM-F12-HAM medium with or without the presence of 250 ng/mL irisin (Ballak et al., 2013; Kristóf et al., 2019). The release of CXCL1, CX3CL1, IL-32, TNFα, and IL1-β were analyzed from the stored samples using ELISA Kits (R&D systems, MN, United States).

### Human Umbilical Vein Endothelial Cell Adhesion Assay

A human umbilical vein endothelial cell (HUVEC) cell line was generated from endothelial cells isolated from the human umbilical cord vein of a healthy newborn by collagenase digestion as described earlier (Palatka et al., 2006). Cells were cultured in M199 medium (Biosera, Nuaille, France) containing 10% FBS (Thermo Fisher Scientific), 10% EGM2 Endothelial Growth Medium (Lonza, Basel, Switzerland), 20 mM HEPES (Biosera), 100 U/mL Penicillin, 100 μg/mL Streptomycin and 2.5 μg/mL Amphotericin B (Biosera), and immortalized by the viral delivery of telomerase gene using pBABE-neo-hTERT (Counter et al., 1998) (gift from Bob Weinberg, 1774, Addgene). The virus packaging was performed in HEK293FT cells (Thermo Fisher Scientific) based on a calcium precipitation method using pUMVC and pCMV-VSV-G vectors (Stewart et al., 2003) (gift from Bob Weinberg, 8449 and 8454, Addgene). The pseudovirion containing supernatant was used for infection, and selection was started 72 h later using 300 μg/mL G418 (Merck-Millipore). Immortalized cells completely retain the morphological properties of primary endothelial cells.

Prior to the adhesion assay, EGM2 was omitted from the standard medium of HUVEC cells and FBS content was decreased to 1% (in which condition cell proliferation is unlikely) for 24 h. 96-well plates (Thermo Fisher Scientific) were precoated with fibronectin (Merck-Millipore) at 1.25 μg/mL concentration in PBS, for 1 h at 37°C and then washed twice with PBS. After centrifugation, trypsinized HUVEC samples were diluted for coating based on counting with three parallels using KOVA Glasstic Slide with Counting Grids (KOVA International, Netherlands). Then cells were plated at 1,000 cells/well density and left to adhere for 2 h in the CO_2_-incubator in the mixture (1:1 ratio) of starvation and conditioned media (incubation period from day 8–12 of differentiation) from SC and DN adipocytes, differentiated in the presence or absence of 250 ng/mL irisin, respectively. Where indicated, recombinant human CXCL1 (275-GR, R&D Systems) was used at 2,500 pg/mL, at the highest observed concentration in media of irisin treated *ex vivo* differentiated adipocytes, in starvation media. Unattached cells were removed by once washing with PBS and adhered cells were incubated with starvation media containing CellTiter-Blue Cell Viability reagent (resazurin; Promega, WI, United States; 36 times dilution). To determine the ratio of attached cells in various conditions, the fluorescent intensity change of each well (Ex:530 nm/Em:590 nm), due to the conversion of resazurin to resorufin by cellular metabolism, was measured using Synergy H1 (BioTek, Hungary) plate reader 2, 4, 6, 18, and 24 h after adding resazurin. Fluorescent intensity values were plotted with respect to time, followed by calculation of slope, which gave the relative adhesion values, after subtraction of values for only starvation media without cells. A linear slope was obtained, which suggests that there could be only negligible cell proliferation, and the gained values represent endothelial cell adhesion measuring the attached viable endothelial cells during the treatments. The final value of adhesion was represented in RFU/hr units and taken from the mean of technical parallels with a minimum of three independent repetitions.

### Statistics and Image Analysis/Preparation

Results are expressed as mean ± SD for the number of independent repetitions indicated. For multiple comparisons of groups, statistical significance was determined by one- or two-way analysis of variance followed by Tukey *post hoc* test. In comparison of two groups, two-tailed unpaired Student’s *t*-test was used. For the design of graphs and evaluation of statistics, Graphpad Prism 9 was used.

## Results

### Irisin Did Not Change the Differentiation Potential of Adipocytes While Increased the Expression of Integrin Receptor Genes in Both SC and DN Origins

**FIGURE 1:**
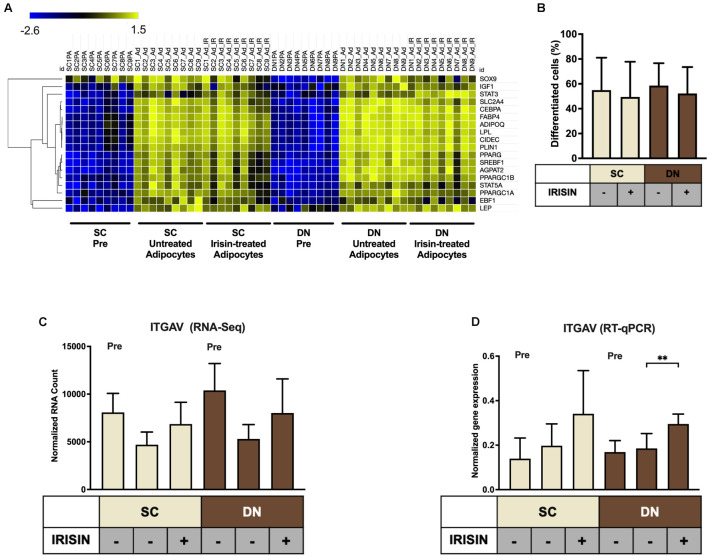
Preadipocytes from subcutaneous (SC) and deep-neck (DN) depots of human neck differentiated at a similar extent irrespective of the presence of irisin. SC and DN preadipocytes (Pre) were differentiated for 2 weeks to white adipocytes. Where indicated, 250 ng/ml irisin was administered during the whole differentiation process. **(A)** Heatmap illustrating the expression of general adipogenic differentiation markers in samples used for Global RNA Sequencing (*n* = 9), **(B)** Quantification of differentiation rate by laser-scanning cytometry (*n* = 9), **(C)** Quantification of *ITGAV* gene expression determined by RNA Sequencing (*n* = 9), and **(D)** RT-qPCR, normalized to *GAPDH* (*n* = 5). Data presented as Mean ± SD. ^∗∗^*p* < 0.01. Statistics: Welch’s *t*-test **(D)**.

Primary hASCs from nine independent donors were isolated and cultivated from SC and DN area of human neck, as described (Tóth et al., 2020). Adipogenic differentiation was driven by a white adipocyte differentiation medium with or without the presence of irisin for 14 days. Then, the global gene expression pattern of differentiated adipocytes and undifferentiated hASCs were determined by global RNA-sequencing (Tóth et al., 2020). Gene expression of general adipocyte markers (e.g., *FABP4*, *ADIPOQ*) was higher in all differentiated adipocytes as compared to preadipocytes (Figure 1A). Quantification of the adipogenic differentiation rate by laser-scanning cytometry (Kristóf et al., 2015) revealed that more than 50% of the cells were differentiated following our 14-days long differentiation protocol (Figure 1B). The presence of irisin did not affect the differentiation and gene expression of general adipocyte markers (Figures 1A,B). A recent publication proposed the receptors for irisin to be integrins, Integrin subunit alpha V (ITGAV) and Integrin subunit beta (ITGB) 1/3/5 (ITGB1/3/5) (Kim et al., 2018). Hence the expression of *ITGAV* was analyzed from RNA-sequencing data (Figure 1C), which revealed that it is expressed in both the preadipocytes and differentiated adipocytes. Upon RT-qPCR validation, a significant increase of *ITGAV* expression was observed in DN adipocytes in response to irisin (Figure 1D). RNA-sequencing data showed that *ITGB1*, *3*, and *5* were also expressed at a high extent in preadipocytes and in differentiated adipocytes irrespective of the presence of irisin (Supplementary Figure 1).

### Genes Involved in Chemokine Signaling Pathways Were Upregulated in Adipocytes Differentiated With Irisin

**FIGURE 2:**
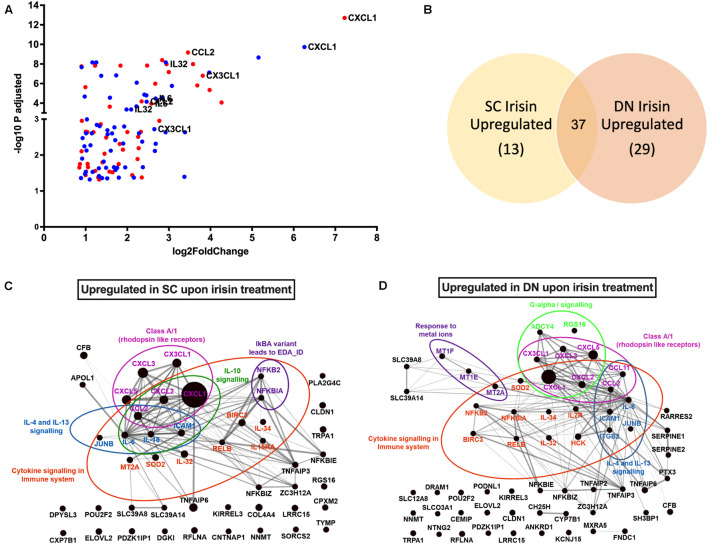
Irisin upregulated similar gene-sets that encode for cytokines in subcutaneous (SC) and deep-neck (DN) depots of human neck area adipocytes. SC and DN preadipocytes were differentiated and treated as in Figure 1. **(A)** Volcano plot showing each of the upregulated genes in SC (red) and DN (blue) depots upon irisin treatment; the highest upregulated genes are listed separately, **(B)** Venn-diagram illustrating the genes commonly upregulated by irisin treatment in SC and DN depots. Gephi illustrations highlighting the most important pathways and the interaction of genes upregulated by irisin treatment in SC **(C)** and DN **(D)** derived adipocytes.

RNA-Sequencing analysis identified 79 genes to be higher expressed upon irisin treatment that are visualized by a Volcano plot (Figure 2A). 50 and 66 genes were significantly upregulated in SC and DN area adipocytes, respectively, each of which are listed in Supplementary Table 3. 37 genes, including *CXCL1*, *CX3CL1*, *IL32*, *IL34*, *IL6*, and *CCL2* were found to be commonly upregulated in adipocytes of both depots (Figures 2A,B and Supplementary Table 3). Surprisingly, thermogenic marker genes did not appear among these. Panther enrichment analysis of genes upregulated in both SC and DN adipocytes by irisin treatment revealed pathways such as cytokine signaling (*NFKB2*, *CXCL1*, *CXCL2*, *IL32*, *IL34*, *IL6*, *CCL2*), interleukin-4 and 13 signaling (*IL6*, *CCL2*, *JUNB*, *ICAM1*), and class A/1 rhodopsin like receptors (*CXCL3*, *CXCL5*, *CX3CL1*, *CXCL2*, *CCL2*, *CXCL1*), which were commonly upregulated in both SC and DN adipocytes (Table 1). Gephi diagrams illustrate the interaction of upregulated genes that belong to several pathways (Figures 2C,D). Interleukin-10 signaling were amongst the upregulated pathways in SC adipocytes (Figure 2C), while in DN, G-alpha-I and response to metal ions were upregulated (Figure 2D). Cluster analyses and heatmap illustration of the gene expression values of the 79 higher expressed genes upon irisin treatment identified two main clusters: a cluster of 25 genes that were uniquely expressed in irisin treated mature adipocytes, and another group of genes that were expressed highly in preadipocytes, but suppressed in differentiated adipocytes without irisin treatment (Supplementary Figure 2). The higher expression of *IL6*, *CCL2*, *CX3CL1*, and *IL32*, cytokine encoding genes was observed by both RNA Sequencing and RT-qPCR analysis (Supplementary Figure 3). Next, we investigated if fractalkine (encoded by *CX3CL1* gene) and IL-32 were released into the conditioned media collected during the differentiation on days numbers 4 and 12; however, we were unable to detect these factors (data not shown).

**TABLE 1:** Pathways of significantly upregulated genes upon irisin treatment during differentiation of subcutaneous (SC) and deep-neck (DN) derived adipocytes.

Panther reactome pathways	Gene name	FDR
**SC Irisin upregulated**		
IkBA variant leads to EDA-ID	***NFKBIA, NFKB2***	4.49 × 10^–2^
Cytokine signaling in immune system	***IL6, NFKBIA, JUNB, IL32, SOD2, MT2A, NFKB2, CXCL2, CCL2*,** *IL15RA*, *IL18*, ***IL34, ICAM1, CXCL1, RELB, BIRC3***	5.23 × 10^–8^
Interleukin-10 signaling	***IL6, CXCL2, CCL2*,** *IL18*, ***ICAM1, CXCL1***	1.65 × 10^–6^
Class A/1 (Rhodopsin like receptors)	***CXCL3, CXCL5, CX3CL1, CXCL2, CCL2, CXCL1***	3.5 × 10^–2^
Interleukin-4 and Interleukin-13 signaling	***IL6, JUNB, CCL2*,** *IL18*, ***ICAM1***	2.3 × 10^–3^
**DN Irisin Upregulated**		
Response to metal ions	***MT2A*,** MT1E, MT1F	4.74 × 10^–3^
Class A/1 (Rhodopsin like receptors)	*CCL11*, ***CXCL3, CXCL5, CX3CL1, CXCL2, CCL2, CXCL1***	1.85 × 10^–2^
Cytokine signaling in immune system	***IL6*,** *CCL11*, *ITGB2*, ***NFKBIA, JUNB, IL32, SOD2, MT2A, NFKB2*,** *IL7R*, ***CXCL2, CCL2, IL34, ICAM1*,** *HCK*, ***CXCL1, RELB, BIRC3***	5.55 × 10^–8^
Interleukin-4 and Interleukin-13 signaling	***IL6*,** *CCL11*, *ITGB2*, ***JUNB, CCL2, ICAM1***	6.33 × 10^–4^
G-alpha (i) signaling events	***CXCL3, CXCL5, CX3CL1*,** *ADCY4*, *RGS16*, ***CXCL2, CXCL1***	5.07 × 10^–2^

*Genes commonly upregulated in both SC and DN area adipocytes are in bold character.*

*CXCL1 was the highest upregulated gene in both SC and DN area adipocytes.*

*FDR, false discovery rate.*

### Irisin Dependent Induction of CXCL1 Release Occurred Predominantly From Differentiating and Mature Adipocytes

**FIGURE 3:**
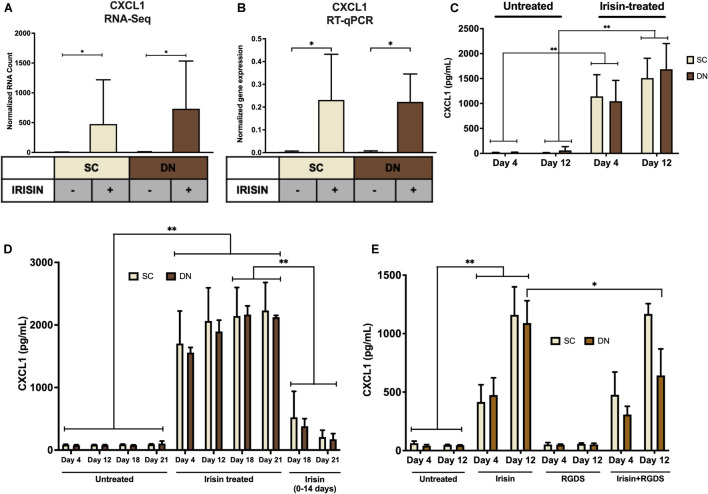
Irisin dependent CXCL1 release was stimulated from differentiating subcutaneous (SC) and deep-neck (DN) area adipocytes. SC and DN preadipocytes were differentiated and treated as in Figures 1, 2. Where indicated, irisin was omitted from the differentiation medium at day 14. Conditioned differentiation media was collected and secreted CXCL1 was measured by sandwich ELISA. **(A)** Quantification of *CXCL1* gene expression as determined by RNA Sequencing (*n* = 9) or RT-qPCR **(B)** normalized to *GAPDH* (*n* = 5), **(C)** CXCL1 release by *ex vivo* differentiating SC and DN adipocytes into the conditioned media collected at the indicated intervals, in the presence or absence of irisin (*n* = 4), **(D)** CXCL1 release in conditioned medium collected at indicated intervals from untreated (21 days) and irisin treated (14 and 21 days as indicated) cell-culture samples (*n* = 3), **(E)** CXCL1 release from differentiating adipocytes with or without irisin treatment, in the presence or absence of 10 μg/ml RGDS (*n* = 4). Comparisons are for the respective days in case of ELISA. Data presented as Mean ± SD. ^∗^*p* < 0.05, ^∗∗^*p* < 0.01. Statistics: GLM **(A)**, One-way ANOVA with Tukey’s post-test **(B)**, Two-way ANOVA with Tukey’s post-test **(C–E)**.

Irisin upregulated *CXCL1* gene expression at the largest extent in both SC and DN area adipocytes (Figures 2A, 3A and Supplementary Table 3). This observation was verified by RT-qPCR (Figure 3B). As a next step, release of CXCL1 from irisin treated and untreated adipocytes was investigated into the conditioned differentiation media collected on the fourth and twelfth days of differentiation. Irisin treatment resulted in significant increase in CXCL1 secretion at the intervals of days 0–4 and 8–12 in both types of adipocytes (Figure 3C).

We aimed to further investigate the dependence of CXCL1 release on the presence of irisin. Therefore, we differentiated hASCs for 21 days, with three sets of samples, each from SC and DN derived adipocytes. Two sets of hASCs were differentiated as previously described, and for the third set, irisin treatment was discontinued after 14 days. Conditioned media were collected on days number 4, 12, 18, 21 and measured for the release of CXCL1. Large amounts of CXCL1 were secreted throughout the differentiation period in the presence of irisin; however, discontinuation of irisin administration led to gradual and significant reduction of the released chemokine (Figure 3D).

A recent publication indicated that RGDS peptide, an integrin receptor inhibitor, can potentially inhibit the effect of irisin (Kim et al., 2018). Hence, we checked the effect of this peptide on the release of CXCL1 on top of irisin treatment. RGDS partially reduced the irisin-stimulated release of CXCL1 by DN adipocytes at day 12 of the differentiation period (Figure 3E).

**FIGURE 4:**
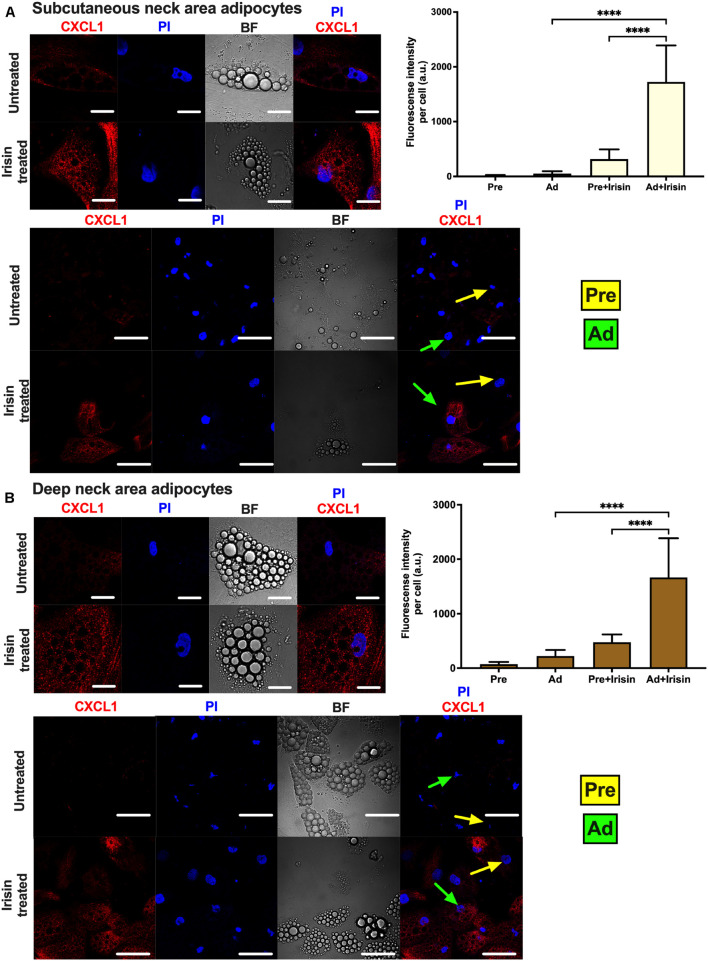
Irisin stimulated CXCL1 release predominantly from subcutaneous **(A)** and deep-neck **(B)** area differentiated adipocytes. Preadipocytes (Pre) were plated and differentiated into adipocytes (Ad) on Ibidi chambers, with or without irisin treatment as in Figures 1–3. Cells were treated with 100 ng/ml brefeldin-A for 24 h to block the secretion of CXCL1, which was followed by fixation and image acquisition by confocal microscopy. Propidium Iodide (PI) was used to stain the nucleus. BF represents the bright field image. Confocal images of differentiated adipocytes were shown followed by wider coverage of undifferentiated and differentiated adipocytes. Scale bars represent 10 μm for single differentiated Ad and 30 μm for wider coverage of Pre and Ad. Yellow and green arrows point the undifferentiated preadipocytes and the differentiated adipocytes, respectively. Quantification of fluorescence intensity normalized to per cell are shown on the right bar graphs. Data presented as Mean ± SD. *n* = 35 cells **(A)** and 50 cells **(B)** from two independent donors. ^****^p < 0.0001. Statistics: One-way ANOVA with Tukey’s post-test.

Release of CXCL1 throughout the whole differentiation period raised a possibility that both undifferentiated preadipocytes and differentiated adipocytes are able to release the chemokine. To investigate this, the secretion machinery of the mixed cell population was inhibited by Brefeldin A, followed by CXCL1 immunostaining and image acquisition by confocal microscopy. Irisin treatment significantly increased CXCL1 immunostaining intensity in both SC (Figure 4A) and DN adipocytes (Figure 4B). Irisin treated adipocytes accumulated significantly more CXCL1 compared to their preadipocyte counterparts in both SC (Figure 4A) and DN areas (Figure 4B). A test for the secondary antibody alone confirmed that the applied secondary antibody did not produce a labeling on its own by unspecifically binding to the cells (Supplementary Figure 4). Our data suggests that irisin stimulates the release of CXCL1 from differentiating and mature adipocytes which is strongly dependent on the presence of irisin but not prominently on its presumed integrin receptor.

### Irisin Stimulates the Release of CXCL1 *via* the Upregulation of NFκB Pathway

**FIGURE 5:**
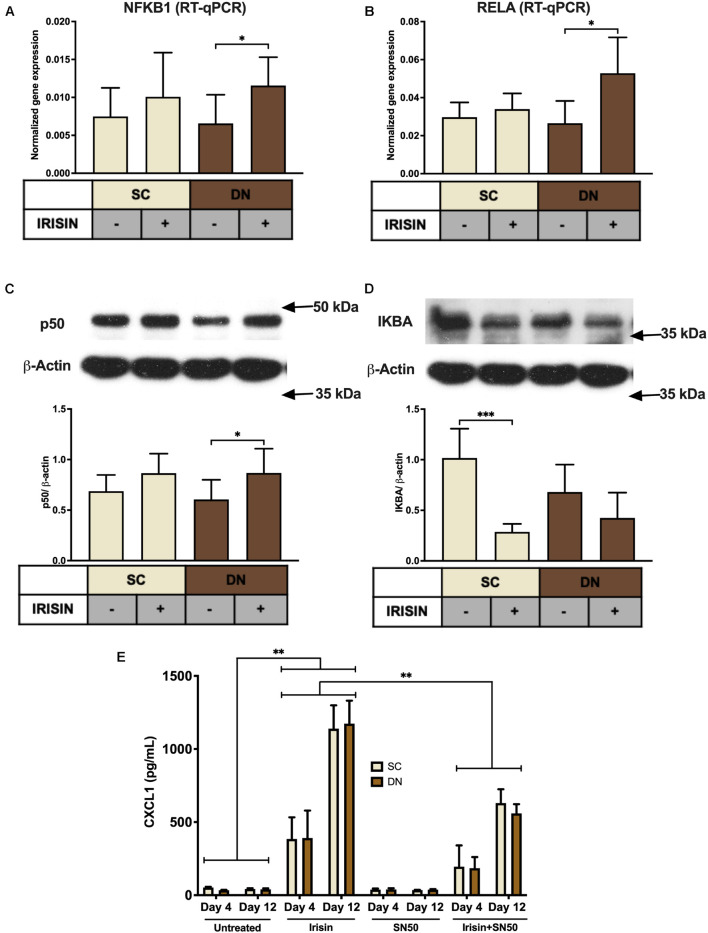
CXCL1 release is stimulated *via* the NFκB pathway during the differentiation of subcutaneous (SC) and deep-neck (DN) area adipocytes. SC and DN preadipocytes were differentiated and treated as in Figures 1–4. Quantification of gene expression for *NFKB1* **(A)** and *RELA* **(B)**, normalized to *GAPDH* by RT-qPCR (*n* = 5), **(C)** p50 and IKBA **(D)** protein expression, normalized to β-actin (*n* = 6), **(E)** CXCL1 release from differentiating adipocytes with or without irisin treatment, in the presence or absence of 50 μg/ml SN50 (*n* = 4); comparisons are for the respective days. Data presented as Mean ± SD. ^∗^*p* < 0.05, ^∗∗^*p* < 0.01, and ^∗∗∗^*p* < 0.001. Statistics: One-way ANOVA with Tukey’s post-test **(A–D)** and Two-way ANOVA with Tukey’s post-test **(E)**.

Next, we aimed to investigate the molecular mechanisms underlying the irisin-induced CXCL1 release. According to our RNA Sequencing data, irisin treatment resulted in a significant upregulation of *NFKB2* and a very modest trend for an increase in *NFKB1* and *RELA* (Supplementary Figures 5A–C) genes. RT-qPCR validation indicated significant upregulation of *NFKB1* (p50 subunit) and *RELA* (p65 subunit) in DN, while an increasing trend was observed in SC adipocytes (Figures 5A,B). p50 protein expression was significantly increased in DN and a slightly increasing trend was found in the case of SC adipocytes (Figure 5C). Protein expression of IκBα, the inhibitor of NFκB transcription factor, decreased significantly upon irisin treatment in SC and a decreasing trend was observed in DN adipocytes (Figure 5D), indicating the upregulation of NFκB pathway.

To prove the direct involvement of the NFκB pathway in adipocyte response to irisin, we applied a cell permeable inhibitor of NFκB nuclear translocation, SN50 (Sárvári et al., 2015), which significantly reduced the release of the chemokine from both types of adipocytes, when it was applied on top of irisin on both the fourth and twelfth days of differentiation, as compared to cells stimulated only by irisin (Figure 5E).

The observed effects of irisin are not likely to be caused by any contamination of endotoxins, which is proved by the negligible expression of *TNF*α or *CCL3* genes (Supplementary Figures 5D,E), and the decreasing trend of *IL1*β gene expression (Supplementary Figure 5F) in irisin treated adipocytes. Furthermore, we did not detect secreted TNFα or IL-1β in the conditioned media of either untreated or irisin treated SC and DN derived adipocytes (data not shown).

### CXCL1 Released From Irisin Stimulated Adipocytes and Adipose Tissue Improves the Adhesion Property of Endothelial Cells

**FIGURE 6:**
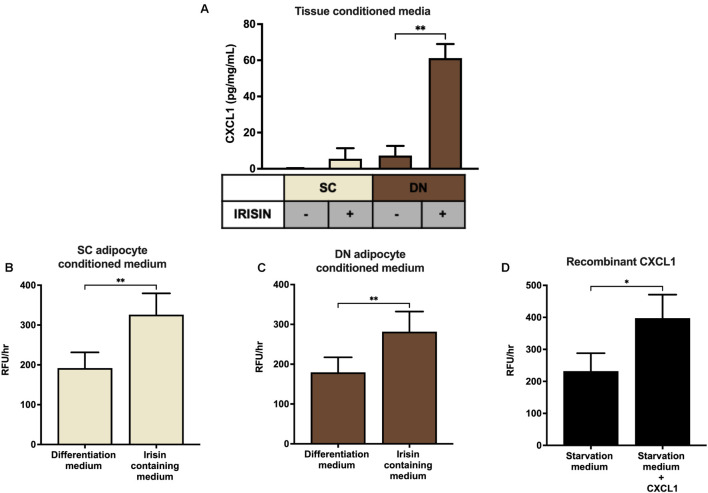
Irisin stimulated the release of CXCL1 from deep-neck (DN) tissue biopsies, which improves the adhesion property of endothelial cells. **(A)** CXCL1 released into the conditioned media of paired subcutaneous (SC) and DN biopsies after 24 h incubation in the presence or absence of irisin (*n* = 4), Quantification of adhesion of endothelial cells upon incubation with the conditioned media (with or without irisin treatment) from *ex vivo* differentiated (incubation period from day 8–12 of differentiation) SC **(B)** and DN **(C)** area adipocytes (*n* = 5), **(D)** Quantification of endothelial cell adhesion upon incubation with recombinant CXCL1 in starvation medium (*n* = 3). Data presented as Mean ± SD. ^∗^*p* < 0.05, ^∗∗^*p* < 0.01. Statistics: One-way ANOVA with Tukey’s post-test **(A)** and Welch’s *t*-test **(B–D)**.

Finally, SC and DN paired tissue biopsies were floated in the presence or absence of irisin dissolved in empty media, followed by quantification of CXCL1 release. The secretion of the chemokine was significantly stimulated from DN tissue biopsies upon irisin treatment (Figure 6A).

Secretion of CXCL1 plays an important role in wound repair and angiogenesis (Gillitzer and Goebeler, 2001). Angiogenesis is crucial for the thermogenic function of BAT (Cannon and Nedergaard, 2004). Therefore, we intended to detect whether the released chemokine can contribute to increased adhesion ability of endothelial cells. Conditioned media collected on the twelfth day of *ex vivo* differentiation, from untreated and irisin treated SC and DN area adipocytes, were added to HUVECs followed by a resorufin based adhesion assay. The conditioned medium from irisin treated adipocytes, which contains various released factors (including CXCL1) was able to significantly increase the number of attached viable HUVECs, compared to the conditioned medium of untreated adipocytes (Figures 6B,C). When HUVECs were treated with recombinant CXCL1, at the highest observed concentration in media of irisin treated *ex vivo* differentiated adipocytes, their adhesion property was enhanced significantly (Figure 6D). This suggests a potential beneficial role of the released CXCL1 in promoting endothelial functions and adipose tissue remodeling to support efficient thermogenesis indirectly by enhancing vascularization.

## Discussion

Irisin was discovered as a proteolytic product of FNDC5, released by cardiac and skeletal myocytes, which induces a beige differentiation program in mouse subcutaneous WAT (Boström et al., 2012; Aydin et al., 2014). In humans, adenine has been shown to be replaced by guanine in the start codon of the human *FNDC5* gene, which was shown to result in a shorter precursor protein lacking the part from which irisin is cleaved (Raschke et al., 2013). Despite this, the presence of irisin in human blood plasma could be detected using mass spectrometry or different antibodies at 3–4 ng/mL (Jedrychowski et al., 2015). The reported concentration range, however, is subject to uncertainty even according the authors themselves, who discussed that they could not account for how much irisin was lost during sample preparation (Jedrychowski et al., 2015). A recent publication indicated the level of circulating irisin in mice to be 0.3 ng/mL, which was previously estimated to be 800 ng/mL (Maak et al., 2021). Furthermore, it is present in the cerebrospinal fluid, liver, pancreas, stomach, saliva, and urine (Mahgoub et al., 2018). However, further research and validated commercially available techniques are required to assess the irisin concentration of human samples in a reproducible manner.

The applied concentrations and time intervals of recombinant irisin largely vary in the experiments reported. The effect of irisin has been intensively studied in various cellular models before any measurement of the hormone level in a physiological context was successfully carried out. In several studies, the recombinant peptide was applied at higher concentrations than its reported range in human plasma (Jedrychowski et al., 2015). Of note, the biological activity of commercially available recombinant peptides might be less than the endogenous hormone, as a result of folding deficiency, partial denaturation or lack of possible post-translational modifications. Irisin significantly increased UCP1 gene and protein expression of rat primary adipocytes at concentrations from 2 to 100 nM that corresponds to 25–1,250 ng/mL (Zhang et al., 2014). The expression of BAT marker proteins (PGC1α, PRDM16, and UCP1) was increased when the peptide was applied at 20 nM (250 ng/mL) on 3T3L1 adipocytes (Tsai et al., 2020). Irisin also protected murine osteocyte-like cells from hydrogen peroxide induced apoptotic cell death at concentrations up to 500 ng/mL (Kim et al., 2018). Irisin elevated mitochondrial respiration of human visceral and subcutaneous WAT-derived and perirenal BAT-derived adipocytes when applied at 50 nM (625 ng/mL) (Li et al., 2019). Another study reported that irisin treatment induced UCP1 protein expression in subcutaneous human adipocytes when the peptide was applied at 50 nM (Huh et al., 2014). Mediastinal brown hASCs that were directionally differentiated in the presence of FNDC5 at 20 nM (800 ng/mL) exhibited a higher gene expression profile of brown marker genes as compared to the untreated cells (Silva et al., 2014). We reported that recombinant irisin at above 50 ng/mL induced a beige phenotype of human primary abdominal subcutaneous and Simpson-Golabi-Behmel syndrome (SGBS) adipocytes when they were treated on top the white adipogenic protocol that was used in this study (Kristóf et al., 2015; Klusóczki et al., 2019). In our previous experiments, irisin administration at 250 ng/mL also facilitated the secretion of batokines, such as IL-6 and MCP1, by abdominal subcutaneous and neck area adipocytes (Kristóf et al., 2019).

Adipocytes from the neck, especially the DN, area play a significant role in maintaining whole body energy homeostasis by performing continuous non-shivering thermogenesis (Svensson et al., 2011; Wu et al., 2012; Cypess et al., 2013; Jespersen et al., 2013). However, the effect of irisin during the differentiation of SC and DN area adipocytes has not yet been elucidated. Recent publications pointed out that irisin may induce a different degree of browning response based on the origin of the human adipose tissue (Buscemi et al., 2018; Li et al., 2019). According to our RNA-sequencing results presented here, irisin did not directly influence the expression of thermogenesis-related genes in the SC and DN area adipocytes. However, it induced components of a secretory pathway leading to the release of *CXCL1*.

The targeted genetic impairment of the thermogenic capacity of BAT in mice (e.g., Ucp1^–/–^ mice) results in a less pronounced phenotype than the ablation of BAT (Villarroya et al., 2019). Transplantation of small amounts of BAT or activated beige adipocytes leads to significant effects on systemic metabolism, including increased glucose tolerance or attenuated fat accumulation in the liver in response to an obesogenic diet (Min et al., 2016). Further studies highlighted the important secretory role of BAT, leading to an increased interest in identifying batokines in rodents that can exert autocrine, paracrine, or endocrine effects. Several recently discovered batokines, such as FGF21, NRG4, BMP8b, CXCL14, or adiponectin have been shown to exert a protective role against obesity by enhancing beiging of WAT, lipolysis, sympathetic innervation, or polarization of M2 macrophages (Ahmad et al., 2021). We found that IL-6, released as a batokine, directly improves browning of human abdominal subcutaneous adipocytes (Kristóf et al., 2019). Our findings suggest that CXCL1 is a novel adipokine, which can be secreted in response to specific cues. This is further supported by gene expression data from single cell analysis of human subcutaneous adipocytes; in thermogenic cells, genes of *CXCL1*, and other secreted factors, such as *CXCL2*, *CXCL3*, *CXCL5*, *CCL2*, and *IL6*, were significantly upregulated in response to forskolin that models adrenergic stimulation of heat production (Min et al., 2019).

CXCL1 is a small peptide belonging to the CXC chemokine family. Upon binding to its receptor, CXCR2 (Silva et al., 2017), it acts as a chemoattractant of several immune cells, especially neutrophils (Schumacher et al., 1992). CXCL1 initiates the migration of immune and endothelial cells upon injury-mediated tissue repair (Gillitzer and Goebeler, 2001). Conditioned medium containing CXCL1, collected during differentiation of SC and DN adipocytes in the presence of irisin, significantly improved the adhesion property of HUVECs. We observed the similar response when they were directly treated with the recombinant chemokine (Figure 6D). Together this raised a possible beneficial paracrine role of the released CXCL1 from differentiating adipocytes upon irisin treatment, which can be further proven by applying a neutralizing antibody against the chemokine or its receptor. Of note, significant involvement of other released factors cannot be excluded.

Our study shed light on an important role of irisin, as a regulator of cytokine release from differentiating adipocytes of the neck area. The study also indicated the upregulation of various other cytokines, such as *CX3CL1*, *IL32*, *CXCL2*, *IL34*, *CXCL5*, and *CXCL3*. Release of IL-6 and MCP1, encoded by *CCL2*, was detected from media collected during differentiation and was found to be specifically released by differentiated lipid laden adipocytes as described in our previous publication (Kristóf et al., 2019). Further studies are required to reveal the impact of irisin stimulated release of other cytokines, which may have beneficial effects on local tissue homeostasis or metabolic parameters of the entire body.

Irisin can exert non-thermogenic effects on several tissues, including the liver (Tang et al., 2016), central nervous system (Ferrante et al., 2016; Zsuga et al., 2018), blood vessels (Han et al., 2015), or the heart (Xie et al., 2015). In mouse osteocytes, irisin acts *via* a subset of integrin receptor complexes, which are assembled from ITGAV and either ITGB1, ITGB3, or ITGB5 (Kim et al., 2018). These integrins transmit the effect of irisin in inguinal fat and osteoclasts *in vivo* (Kim et al., 2018; Estell et al., 2020). In our experiments, RT-qPCR analysis of *ITGAV* expression has revealed its high expression in both preadipocytes and differentiated adipocytes, which was further upregulated upon irisin treatment in DN adipocytes (Figure 1D). RNA Sequencing also proved that the β-integrin subunits were abundantly expressed in both preadipocytes and differentiated adipocytes (Supplementary Figure 1). However, RGDS peptide exerted only a moderate effect on the irisin-stimulated CXCL1 secretion by DN adipocytes. This suggests that irisin initiates some of its biological effects *via* other, currently unknown receptor(s) as well. The canonical integrin signaling includes the phosphorylation of FAK and Zyxin, followed by phosphorylation of AKT (at T308) and CREB (Kim et al., 2018). However, other studies proposed positive effects of irisin on cAMP-PKA-HSL (Xiong et al., 2015), AMPK (So and Leung, 2016; Xin et al., 2016), or p38 MAPK (Zhang et al., 2014) pathways. Of note, RGDS peptide was applied at a relatively low concentration, in which anoikis was not observed. It is still possible that some of the administered irisin still access their integrin receptors at this condition.

It has already been reported that *CXCL1* gene expression is directly controlled by NFκB (Burke et al., 2014). NFκB-signaling might be induced in *ex vivo* differentiated adipocytes by released saturated fatty acids that can activate toll-like receptor (TLR) 4, which is abundantly expressed at mRNA level in hASCs and adipocytes of human neck (data not shown) (Lee et al., 2003; Suganami et al., 2007). Our data indicate that genes of canonical NFκB-signaling, which are abundantly expressed in neck area adipocytes, are upregulated when differentiated in the presence of irisin (Figures 5A,B). The induced expression of inflammation-related genes might explain why thermogenic genes were not upregulated further when adipocytes were differentiated in the presence of irisin (Chung et al., 2017). The absence of TNFα or IL-1β-upregulation and release during the differentiation in the presence of irisin excluded the possibility of endotoxin contamination of the recombinant hormone. Although irisin was reported previously to inhibit LPS-induced NFκB activation (Mazur-Bialy et al., 2017; Jiang et al., 2020), adipocytes differentiated in the presence of both SN50 and irisin released less CXCL1 than those of treated with irisin alone (Figure 5E). Further research is needed to explore the irisin-induced molecular events in the distinct human adipocyte subsets.

In this study, we have shown that irisin applied in a higher concentration than that reported in human blood plasma upregulated the expression of several genes with respect to cytokine signaling in human adipocytes derived from the neck. CXCL1 was upregulated at the greatest extent, at least partially by upregulation of the NFκB pathway, and was proved to be secreted mainly by differentiated adipocytes. Of note, the expression of thermogenesis-related genes were not induced that might be explained by the desensitization of irisin receptors by the high concentration of the hormone. On the other hand, results of *in vitro* endothelial adhesion assay suggested a positive effect of the released chemokine on angiogenesis. Further studies are required to assess how irisin at physiological levels affects thermogenesis and cytokine release of human adipocytes.

## Data Availability Statement

The datasets presented in this study can be found in online repositories. The names of the repository/repositories and accession number(s) can be found below: https://www.ncbi.nlm.nih.gov/, PRJNA607438.

## Ethics Statement

The studies involving human participants were reviewed and approved by Medical Research Council of Hungary (20571-476 2/2017/EKU). The patients/participants provided their written informed consent to participate in this study.

## Author Contributions

LF, EK, AS, and RK conceived and designed the experiments. AS, EK, SP, RK, and AV performed the experiments. EK, AS, and AV generated primary cell cultures for the experiments. BT analyzed the RNAseq data. RA analyzed and visualized gene interaction networks. IC, AS, AV, and ZB performed microscopy and image analysis. FG provided tissue samples, IK-S provided HUVEC cells. AS and EK wrote the manuscript with inputs from BT. LF mentored the writing and revised the draft. LF, EK, and IK-S acquired funding. All authors approved the submitted version.

## Conflict of Interest

The authors declare that the research was conducted in the absence of any commercial or financial relationships that could be construed as a potential conflict of interest.

## Publisher’s Note

All claims expressed in this article are solely those of the authors and do not necessarily represent those of their affiliated organizations, or those of the publisher, the editors and the reviewers. Any product that may be evaluated in this article, or claim that may be made by its manufacturer, is not guaranteed or endorsed by the publisher.

## Footnotes

1
https://pantherdb.org

2
https://software.broadinstitute.org/morpheus

3
https://string-db.org

4
https://gephi.org

## Abbreviations

BAT: brown adipose tissue
CXCL: C-X -C motif chemokine ligand
DN: deep-neck derived adipocytes
GRO: growth-related oncogene
hASCs: human adipose-derived stromal cells
HUVEC: human umbilical vein endothelial cells
IgG: immunoglobulin G
IL: interleukin
MCP1: monocyte chemoattractant protein 1
NF κ B: nuclear factor- κ B
PI: propidium iodide
SC: subcutaneous neck derived adipocytes
WAT: white adipose tissue.
