# Supplementary material for: Irisin Stimulates the Release of CXCL1 From Differentiating Human Subcutaneous and Deep-Neck Derived Adipocytes via Upregulation of NFκB Pathway
Source: Front Cell Dev Biol. 2021 Oct 11;9:737872. doi: 10.3389/fcell.2021.737872 (PMC8542801; doi:10.3389/fcell.2021.737872)
Supplement: Supplementary file 1 [file Data_Sheet_1.DOCX]

Supplementary Material


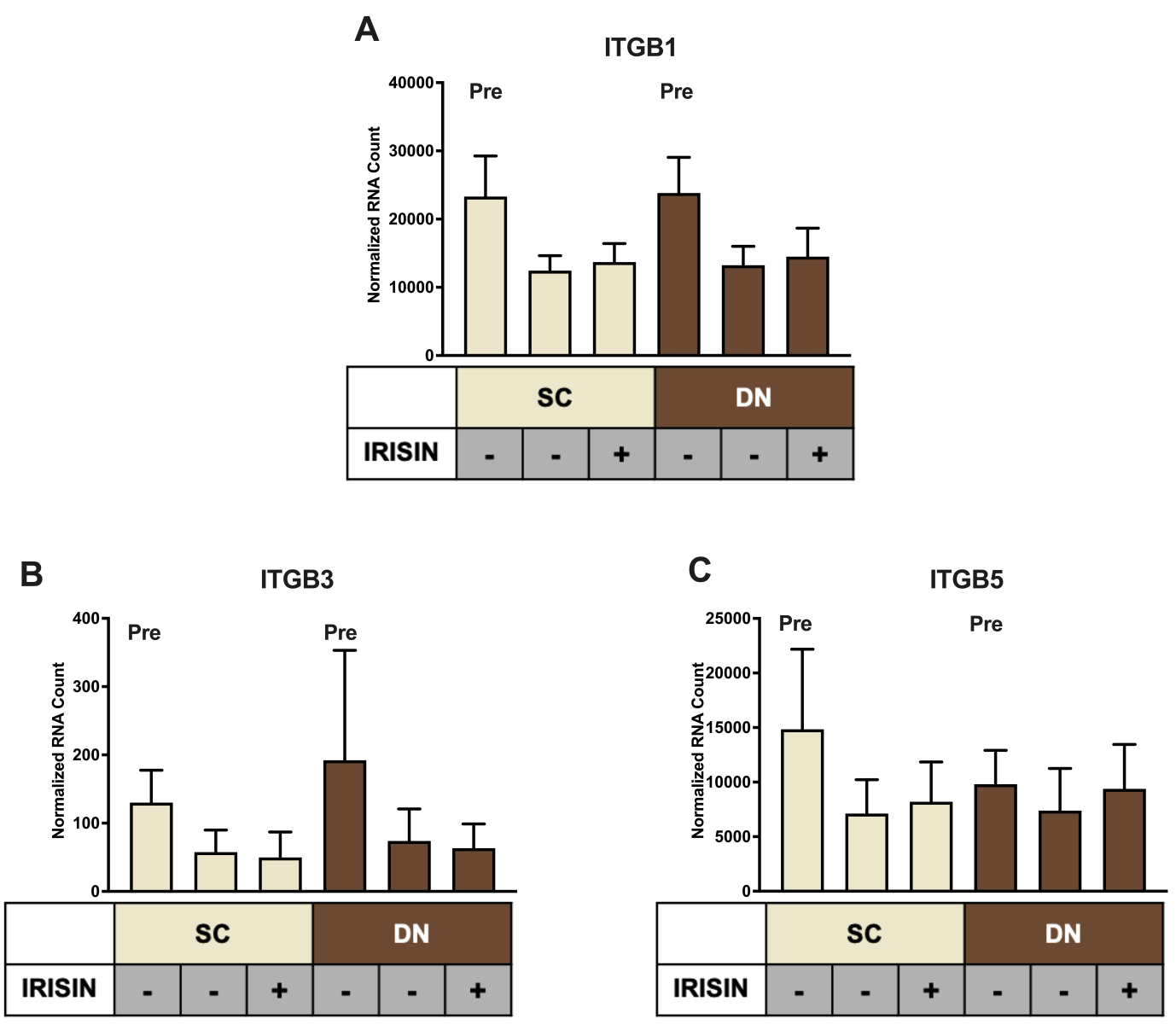


**Supplementary Figure 1.** **Proposed irisin receptor subunits are abundantly expressed in subcutaneous (SC) and deep-neck (DN) preadipocytes (Pre) and adipocytes of human neck.** SC and DN preadipocytes were differentiated and treated as in Figure 1. Quantification of gene expression of *ITGB1* (A), *ITGB3* (B), and *ITGB5* (C) by RNA-Sequencing (n=9). Data presented as Mean ± SD.


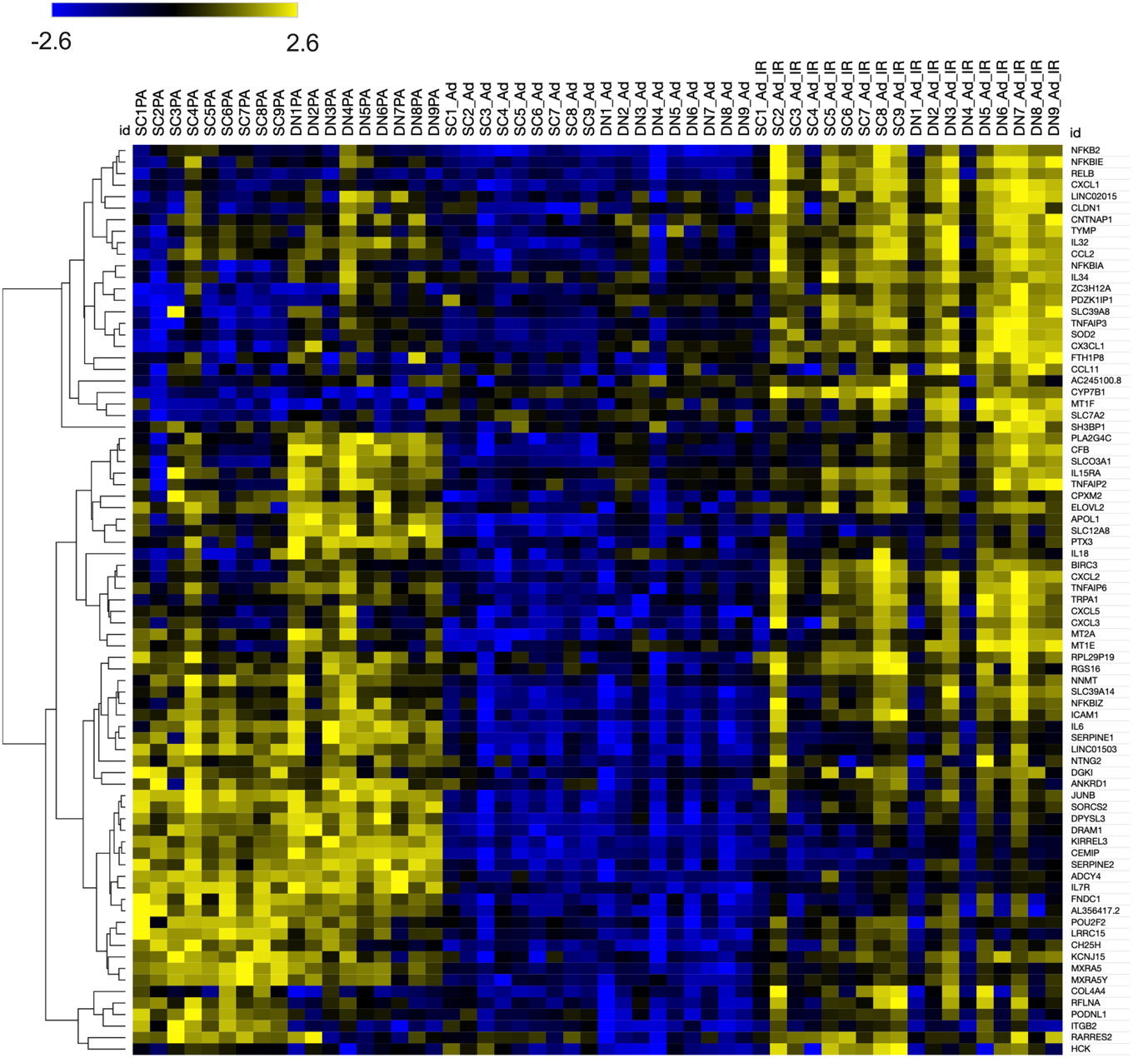


**Supplementary figure 2. Heatmap highlighting genes upregulated by irisin treatment in subcutaneous (SC) and deep-neck (DN) preadipocytes (Pre) and adipocytes (Ad) of human neck.** SC and DN preadipocytes were differentiated and treated as in Figure 1. Heatmap illustrating the expression of genes upregulated by irisin among all samples as evaluated by RNA Sequencing.


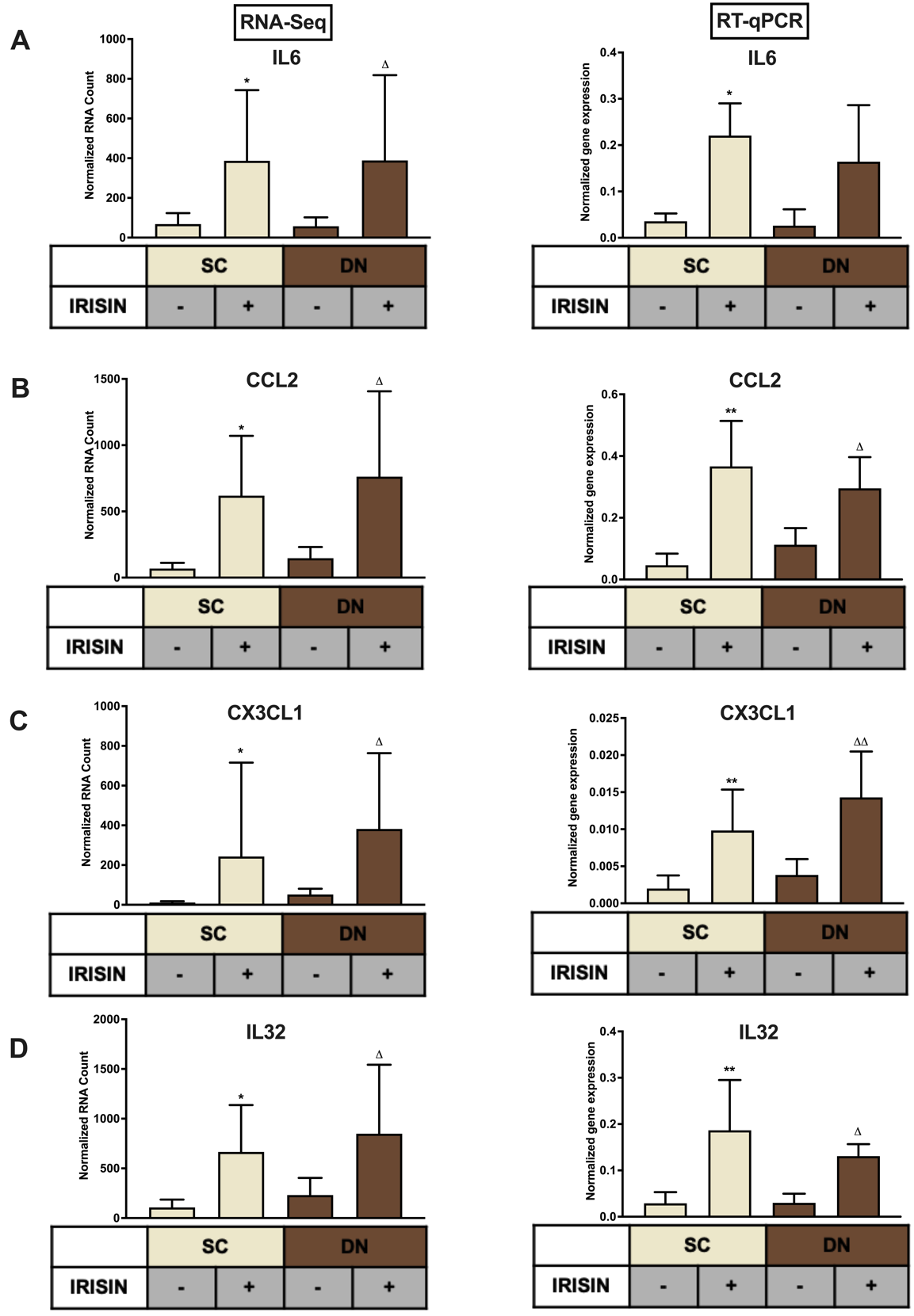


**Supplementary figure 3. Evaluation and validation of RNA Sequencing data for genes encoding cytokines, during the differentiation of subcutaneous (SC) and deep-neck (DN) derived adipocytes upon irisin treatment.** SC and DN preadipocytes were differentiated and treated as in Figure 1. Quantification of gene expression of *IL6* (A), *CCL2* (B), *CX3CL1* (C), and *IL32* (D) as assessed by RNA Sequencing (left, n=9) and RT-qPCR normalized to *GAPDH* (right, n=5). Data presented as Mean ± SD. * : Refers to compared with SC, △ : Refers to compared with DN. *,^△^ p<0.05 and **, ^△△^ p<0.01. Statistics: GLM (RNA-Sequencing) and One-way ANOVA with Tukey’s post-test (RT-qPCR).


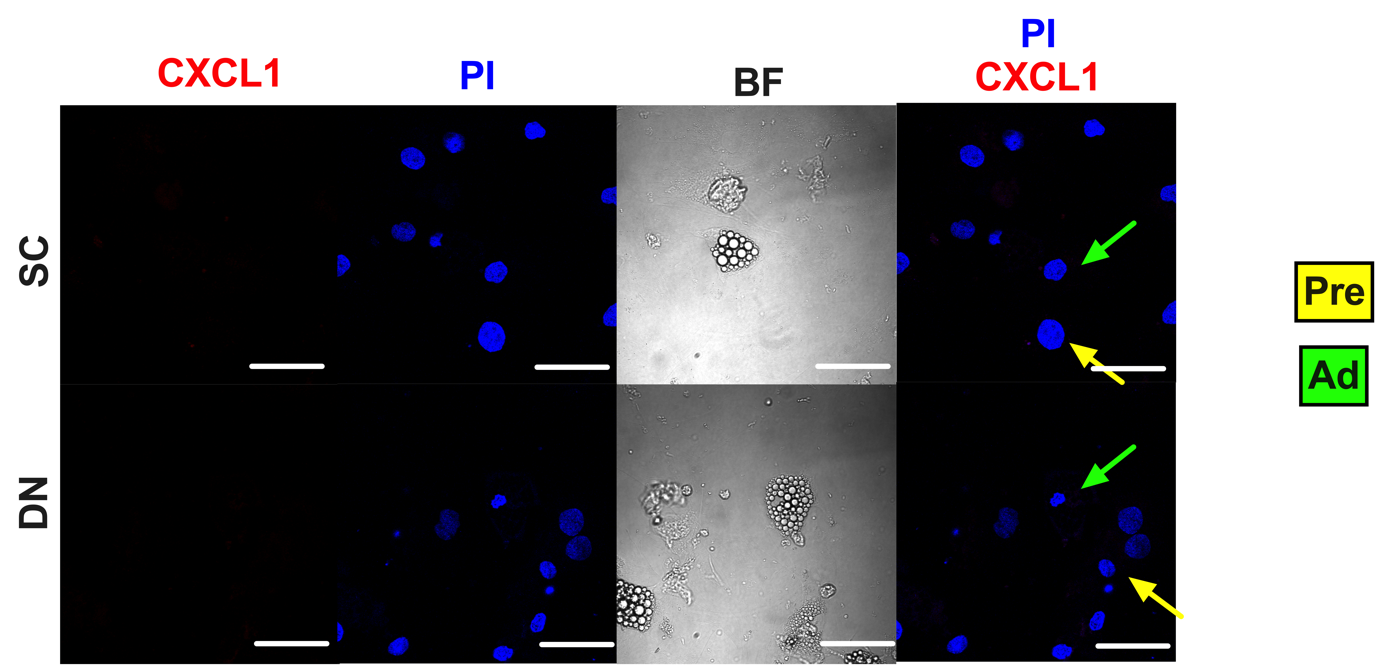


**Supplementary figure 4. Representative images of secondary antibody controls proving the specificity of CXCL1 immunostaining.** Scale bars represent 30 μm.


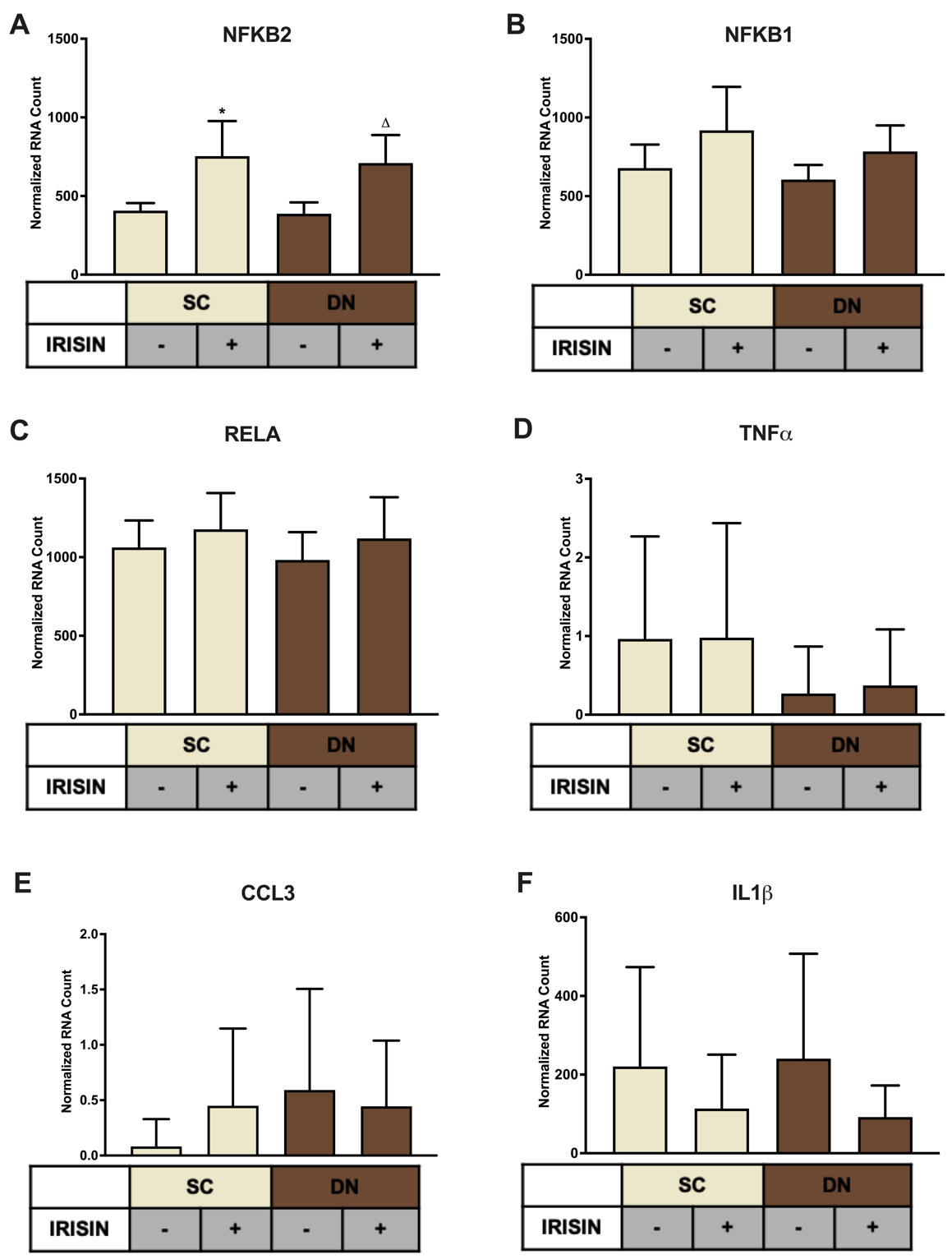


**Supplementary figure 5. Irisin treatment upregulated genes related to NFκB pathway during the differentiation of subcutaneous (SC) and deep-neck (DN) area adipocytes, while the expression of pro-inflammatory markers remained unchanged.** SC and DN preadipocytes were differentiated and treated as in Figures 1-4. Quantification of gene expression of *NFKB2* (A), *NFKB1* (B), *RELA* (C), *TNFα* (D), *CCL3* (E), and *IL1β* (F) as assessed by RNA Sequencing. Data presented as Mean ± SD. * : Refers to compared with SC, △ : Refers to compared with DN, *,^△^ p<0.05, n=9 (Statistics: GLM)

**Supplementary Table 1. Table listing details of donors used for RNA-Sequencing study**

| **Donor number** | **Gender** | **Age** |
| --- | --- | --- |
| 1 | M | 66 |
| 2 | F | 53 |
| 3 | M | 45 |
| 4 | F | 35 |
| 5 | F | 64 |
| 6 | F | 64 |
| 7 | F | 73 |
| 8 | F | 66 |
| 9 | M | 57 |

**Supplementary Table 2. Table listing gene expression assays used in the study**

| **Genes** | **Assay ID** |
| --- | --- |
| *ITGAV* | Hs00233808_m1 |
| *CXCL1* | Hs00236937_m1 |
| *NFKB1* | Hs00765730_m1 |
| *RELA* | Hs00153294_m1 |
| *IL6* | Hs00985639_m1 |
| *CCL2* | Hs00234140_m1 |
| *CX3CL1* | Hs00171086_m1 |
| *IL32* | Hs00992441_m1 |
| *GAPDH* | Hs99999905_m1 |

**Supplementary Table 3. Significantly upregulated genes during the differentiation of subcutaneous (SC) and deep-neck (DN) derived adipocytes upon irisin treatment.**

| **SC Irisin Upregulated** | |  | **DN Irisin Upregulated** | |
| --- | --- | --- | --- | --- |
| **Gene-symbol** | **log2FoldChange** |  | **Gene-symbol** | **log2FoldChange** |
| *CXCL1* | 7.224 |  | *CXCL1* | 6.258 |
| *CXCL3* | 4.266 |  | *CXCL5* | 5.154 |
| *CXCL5* | 3.979 |  | *CXCL2* | 3.970 |
| *CX3CL1* | 3.813 |  | *CXCL3* | 3.387 |
| *CXCL2* | 3.684 |  | *HCK* | 3.374 |
| *TNFAIP6* | 3.581 |  | *TNFAIP6* | 3.073 |
| *CCL2* | 3.460 |  | *CFB* | 2.926 |
| *CFB* | 2.997 |  | *SLC7A2* | 2.922 |
| *IL32* | 2.945 |  | *RFLNA* | 2.847 |
| *BIRC3* | 2.838 |  | *CLDN1* | 2.683 |
| *COL4A4* | 2.771 |  | *IL6* | 2.682 |
| *ICAM1* | 2.673 |  | *CCL11* | 2.664 |
| *IL6* | 2.581 |  | *CX3CL1* | 2.652 |
| *SOD2* | 2.478 |  | *CCL2* | 2.465 |
| *CLDN1* | 2.349 |  | *ICAM1* | 2.460 |
| *TRPA1* | 2.345 |  | *TRPA1* | 2.411 |
| *RFLNA* | 2.327 |  | *MT1F* | 2.375 |
| *LRRC15* | 2.272 |  | *LRRC15* | 2.360 |
| *ELOVL2* | 2.262 |  | *ELOVL2* | 2.282 |
| *IL18* | 2.247 |  | *SOD2* | 2.228 |
| *LINC02015* | 2.119 |  | *MT2A* | 2.220 |
| *MT2A* | 2.002 |  | *AL356417.2* | 2.159 |
| *RGS16* | 1.897 |  | *IL32* | 2.094 |
| *TNFAIP3* | 1.837 |  | *MXRA5Y* | 1.982 |
| *PDZK1IP1* | 1.818 |  | *FNDC1* | 1.950 |
| *CPXM2* | 1.794 |  | *POU2F2* | 1.926 |
| *AC245100.8* | 1.645 |  | *CH25H* | 1.902 |
| *CYP7B1* | 1.579 |  | *ANKRD1* | 1.798 |
| *POU2F2* | 1.572 |  | *RGS16* | 1.757 |
| *SLC39A8* | 1.493 |  | *TNFAIP3* | 1.735 |
| *KIRREL3* | 1.452 |  | *IL7R* | 1.717 |
| *IL34* | 1.405 |  | *BIRC3* | 1.711 |
| *NFKBIZ* | 1.361 |  | *KCNJ15* | 1.691 |
| *DPYSL3* | 1.324 |  | *SERPINE1* | 1.680 |
| *SORCS2* | 1.302 |  | *MXRA5* | 1.635 |
| *APOL1* | 1.228 |  | *PDZK1IP1* | 1.604 |
| *RELB* | 1.227 |  | *NFKBIZ* | 1.583 |
| *TYMP* | 1.187 |  | *KIRREL3* | 1.527 |
| *RPL29P19* | 1.056 |  | *SLC39A8* | 1.523 |
| *IL15RA* | 1.048 |  | *SERPINE2* | 1.518 |
| *NFKBIA* | 1.042 |  | *ITGB2* | 1.490 |
| *NNMT* | 1.009 |  | *ADCY4* | 1.434 |
| *JUNB* | 0.999 |  | *CEMIP* | 1.396 |
| *DGKI* | 0.999 |  | *SLC39A14* | 1.374 |
| *NFKBIE* | 0.995 |  | *LINC01503* | 1.336 |
| *CNTNAP1* | 0.908 |  | *PODNL1* | 1.332 |
| *ZC3H12A* | 0.901 |  | *PTX3* | 1.320 |
| *NFKB2* | 0.899 |  | *IL34* | 1.317 |
| *PLA2G4C* | 0.862 |  | *RPL29P19* | 1.269 |
| *SLC39A14* | 0.849 |  | *RELB* | 1.255 |
|  |  |  | *CYP7B1* | 1.166 |
|  |  |  | *NFKBIE* | 1.162 |
|  |  |  | *MT1E* | 1.150 |
|  |  |  | *NNMT* | 1.115 |
|  |  |  | *SH3BP1* | 1.097 |
|  |  |  | *JUNB* | 1.030 |
|  |  |  | *RARRES2* | 1.020 |
|  |  |  | *TNFAIP2* | 1.010 |
|  |  |  | *DRAM1* | 1.001 |
|  |  |  | *SLC12A8* | 0.986 |
|  |  |  | *SLCO3A1* | 0.977 |
|  |  |  | *NFKBIA* | 0.970 |
|  |  |  | *ZC3H12A* | 0.926 |
|  |  |  | *FTH1P8* | 0.916 |
|  |  |  | *NFKB2* | 0.902 |
|  |  |  | *NTNG2* | 0.888 |
